# Developing a questionnaire to determine the impact of self-management in diabetes: giving people with diabetes a voice

**DOI:** 10.1186/s12955-017-0719-4

**Published:** 2017-07-18

**Authors:** J. Carlton, J. Elliott, D. Rowen, K. Stevens, H. Basarir, K. Meadows, J. Brazier

**Affiliations:** 1 School of Health and Related Research (ScHARR), University of Sheffield, Regent Court, Sheffield, S1 4DA UK; 2 Sheffield Teaching Hospitals NHS Trust, Sheffield, UK; 3 Health Economics Unit, Institute of Applied Health Research, College of Medical and Dental Sciences, IOEM Building, University of Birmingham, Edgbaston, Birmingham, B15 2TT UK; 4 DHP Research & Consultancy Ltd, Bloxham Mill Business Centre, Barford Road, Bloxham, Banbury OX15 4FF UK; 5 Department of Oncology and Metabolism, University of Sheffield, Medical School, Sheffield, S10 2JF UK

**Keywords:** Diabetes mellitus, Self-management, Patient reported outcome measure (PROM), Descriptive system, Content validity, Quality of life

## Abstract

**Background:**

The prevalence of diabetes mellitus (DM) is increasing dramatically, placing considerable financial burden on the healthcare budget of each country. Patient self-management is crucial for the control of blood glucose, which largely determines the chances of developing diabetes-related complications. Self-management interventions vary widely, and a method is required for assessing the impact of self-management. This paper describes the development of a questionnaire intended for use to measure the impact of self-management in diabetes.

**Methods:**

An iterative development process was undertaken to identify the attributes of self-management using 5 steps. First, a literature review was undertaken to identify and understand themes relating to self-management of DM to inform a topic guide. Second, the topic guide was further refined following consultation with a Patient and Public Involvement group. Third, the topic guide was used to inform semi-structured interviews with patients with Type 1 DM (T1DM) and Type 2 DM (T2DM) to identify how self-management of DM affects individuals. Fourth, the research team considered potential attributes alongside health attributes from an existing measure (Diabetes Health Profile, DHP) to produce an instrument reflecting both health and self-management outcomes simultaneously. Finally, a draft instrument was tested in a focus group to determine the wording and acceptability.

**Results:**

Semi-structured interviews were carried out with 32 patients with T1DM and T2DM. Eight potential attributes were identified: fear/worry/anxiety, guilt, stress, stigma, hassle, control, freedom, and feeling supported. Four of these self-management attributes were selected with four health attributes (mood, worry about hypos (hypoglycaemic episodes), vitality and social limitations) to produce the Health and Self-Management in Diabetes (HASMID^v1^) questionnaire.

**Conclusions:**

HASMID^v1^ is a short questionnaire that contains eight items each with four response levels to measure the impact of self-management in diabetes for both T1DM and T2DM. The measure was developed using a mixed-methods approach that involved semi-structured interviews with people with diabetes. The measure has high face validity. Ongoing research is being undertaken to assess the validity of this questionnaire for measuring the impact of self-management interventions in economic evaluation.

**Electronic supplementary material:**

The online version of this article (doi:10.1186/s12955-017-0719-4) contains supplementary material, which is available to authorized users.

## Background

The estimated diabetes mellitus (DM) prevalence worldwide for 2011 was 366 million people and is expected to increase to 552 million by 2030 [1]. These prevalence rates put a considerable financial burden on the healthcare budget of each country. Patient self-management is crucial for the control of blood glucose, which largely determines the chances of developing DM complications over the long-term. Although health care professionals may provide advice and guidance about medications, food intake and the effects of physical activity, the main factors determining success is achieving and maintaining metabolic control are the ability and willingness of the patient to self-manage these tasks themselves [2]. Several large trials on self-management in DM have been conducted [3, 4], and strong evidence exists showing that self-management is essential to maintain ideal blood glucose levels and to avoid long-term diabetes-related events [5].

Cost-effectiveness analyses have been undertaken to examine the impact of self-management on DM in terms of cost per quality adjusted life year (QALY), where quality of life is assessed on a scale from zero for states as bad as being dead to one for full health [6]. These analyses have typically taken the United Kingdom (UK) NHS healthcare perspective, where the within-trial (short-term) and lifetime (long-term) modelling outcomes had focussed strictly on the improvements in clinical outcomes and health-related QALYs, such as the EQ-5D, or used utility estimates obtained for descriptions of the processes of care, such as the insulin regimen, rather than the consequences of self-management on quality of life [7–10]. The use of health related measures like EQ-5D means the day to day consequences of self-management for the life’s of patients is being excluded. The use of specific processes of care limits the ability to compare across different self-management interventions. Furthermore, self-management differs considerably between the two main types of diabetes, partly because the therapies are usually very different. In type 1 diabetes mellitus (T1DM) there is an absolute insulin deficiency so insulin therapy is mandatory from the outset, versus type 2 diabetes mellitus (T2DM) where there is a relative insulin deficiency and a variety of treatments from diet, to tablets, to injectable therapies, may be required depending on the duration and stage of the condition. For either condition, a method is required for assessing the non-health impact of self-management, which is not treatment specific and can be used in different groups of patients and over time. This paper describes the development of a patient-report descriptive system (or questionnaire) that can be used to evaluate the impact of self-management in diabetes for both T1DM and T2DM. The project followed an iterative process, and was informed by patient views throughout, to ensure good face validity.

## Methods

**Fig. 1:**
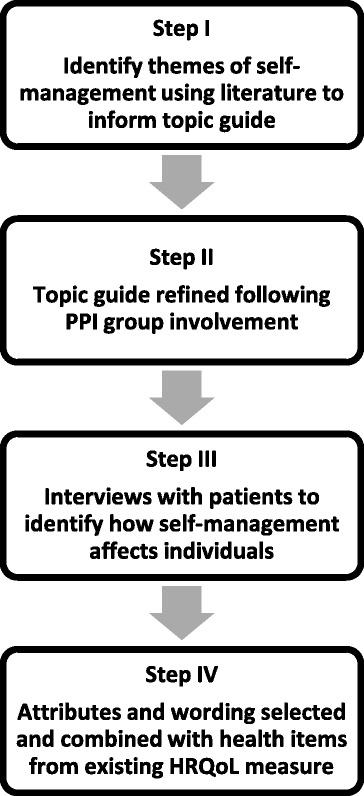
Development of HASMID^v1^ questionnaire

Figure 1 illustrates the process used to develop the questionnaire.

### Step I: Development of a topic guide

A rapid literature review was undertaken to identify and understand themes related to self-management of both T1DM and T2DM to inform a topic guide for the interviews with individuals with T1DM and T2DM. The literature search was conducted at the project outset in May 2014, details of the strategy are shown in Additional file 1: Appendix 1. We selected for review papers published in the last 2 years that reported themes of self-management, which produced 11 papers. All potential themes that were reported are shown in Additional file 1: Appendix 2, and these were used to produce an initial long list of themes for consideration for the topic guide.

### Step II: Topic guide refinement

The potential themes were then considered by the whole research team. Potential themes across both T1DM and T2DM were considered jointly as largely the same themes were raised for both types. Potential themes were excluded if they were based upon health status, health-related questions (i.e., depression), or specifics of a process or intervention (e.g., Insulin regimen, face-to-face or web-based education). The remaining themes were categorised into one of four main overarching categories: task-related, physical, emotional, and social.

Additional themes were added to the topic guide by the research team. These included *information overload*, *reinforcement of behaviours*, *regret* or *guilt*, and *coping*. The potential themes were used to generate a draft topic guide for interviews with patients with diabetes. The draft topic guide was considered by a Patient and Public Involvement (PPI) panel. The panel made a number of suggestions, including the order in which themes could be introduced in the interview and some additional themes to include such as *peer pressure*, *blame* and *self-image* (Additional file 1: Appendix 3). No consensus had to be achieved for an additional theme to be added to the topic guide. If any member of the panel felt an additional theme was necessary this was included. The final lists of themes used in the topic guide are shown in Table 1.

**Table 1:** Themes to be included in topic guide for interviews with patients with DM

Task-related	
Monitoring and burden	
Information overload^a^	
Satisfaction	
Reinforcement of behaviour^a^	
Getting feedback on performance/satisfaction	
Self-efficacy	
Problem solving	
Physical	
Exercise	
Healthy eating	
Being active	
Emotional	
Stress	
Distress	
Denial	
Acceptance	
Regret/guilt^a^	
Confidence	
Coping^a^	
Control	
Empowerment – reduced decision-making	
Blame^a^	
Self-image^a^	
Social	
Support	
Relationship stress	
Peer pressure^a^	
Understanding of others	

^a^additional themes added by PPI panel

### Step III: Interviews with patients: Methods

Semi-structured interviews were conducted with individuals with T1DM and T2DM. The inclusion criteria were that the participant had a clinical diagnosis of DM, over the age of 18 years, and able to provide informed consent. Potential participants who satisfied the inclusion criteria were identified from Sheffield Teaching Hospitals NHS Foundation Trust, a GP practice (which has two sites in Sheffield) and the Sheffield Teaching Hospitals NHS Foundation Trust Research Database for Diabetes, Endocrinology and Retinal Screening. Prior to the interview, each participant had received an information sheet detailing the study. Written consent was obtained prior to the interview being conducted. Following and during the interview, demographic information was obtained to ensure that a balanced sample was achieved for both T1DM and T2DM.

The developed topic guide was used for the interviews and contained themes to be discussed with the participants, instead of a set of specific questions. The aim of the interviews was to identify how self-management of DM affects individuals. Participants were encouraged to talk about issues via open-ended questions, and the individual’s responses were probed to try to identify what aspects of self-management impacted upon their daily lives.

Each interview was digitally recorded, allowing the researcher to devote full attention to the interview itself [11]. The interviews were transcribed verbatim. The transcripts were imported into QSR NVivo 10© (QSR International, Doncaster, Australia), a computer-assisted, qualitative data analysis software package, to manage the data and to facilitate analysis. The analysis was guided by the research question “What are the ways in which self-management impacts on QoL?” The aim of the analysis was to identify how self-management of diabetes impacts upon the individual. Thematic content analysis (where themes are identified in which both the content and the context of the documents are analysed) was undertaken using Framework [11]. Framework follows the principles of classifying and organising data according to key themes, concepts and emergent categories [11]. An initial framework based upon the topic guide was used to guide the analysis, with an emphasis on how self-management of DM impacts upon the individual. Specific process-related attributes of self-management were not included, as this would potentially limit the findings of this study to specific self-management interventions. Instead the research aim was to identify attributes related to how self-managing DM impacts upon an impact upon an individual, which could be subject to change following a self-management intervention (i.e., feeling more in control of their DM). Each transcript was reviewed several times in order to become familiar with the data. Key phrases, sentences and words were identified that related to self-management of diabetes. Any emergent themes were identified. The transcripts were then re-examined and coded according to the identified themes. Once the transcripts were coded into themes, the data was organised into potential attributes that were considered for the questionnaire. Transcripts for both T1DM and T2DM were considered together.

## Interviews with patients: Results

### Patient sample characteristics

All interviews were conducted between 3rd October 2014 and 24th November 2014. Thirty-two participants were interviewed (T1DM *n* = 16, T2DM *n* = 16). One of the interviews was terminated early, as the participant gave limited one-word answers, despite attempts to encourage more explanation behind their responses. Table 2 show a summary of the characteristics of the study population. The sample covers all the main groups of age, gender, HbA1c, age at diagnosis and duration of DM. There was a good spread between T1DM and T2DM. Interviews varied in length from approximately 12 to 64 min, with an average interview length of 32 min. Recruitment continued until data saturation was reached, and the number of interviews conducted exceeded this point. Confidence that data saturation was achieved was high, as all interviews were conducted by one researcher (JC).

**Table 2:** Characteristics of the study population

DM Type	Age (years)							Sex (M)	Ethnicity		Age at diagnosis (years)						HbA1c			Duration of DM (years)					
											T1			T2						T1			T2		
	<20	20–30	31–40	41–50	51–60	61–70	>70		White	Non-white	<20	20–40	>40	<40	40–60	>60	<7.5%	7.5–10%	>10%	<10	10–20	>20	<5	5–15	>15
T1	0	1	1	4	8	0	2	6	16	0	6	6	4	-	-	-	1	12	3	5	2	9	-	-	-
T2	0	1	0	1	1	4	9	10	12	4	-	-	-	3	11	2	4	11	1	-	-	-	1	9	6

### Potential attributes for the descriptive system

Analysis of the qualitative data identified eight potential attributes to be considered for the descriptive system.

#### Fear/Anxiety

Participants discussed emotional responses to having DM, and/or self-managing the condition. Fears, worries and anxieties were linked with a number of components of DM and self-management. Some fears were associated with DM treatment, particularly with insulin. These included a fear of needles, and a fear of being on insulin and the implications that this would have on the individual. Participants also discussed fears or worries of not administering insulin correctly. Such anxieties did not seem to be associated with the length of time that the individual had been on a given type of medication. Individuals still felt uncertain they were doing things as they should, and had a persistent low level of worry despite being on the same medication pathway for a prolonged time period. Some participants spoke of a worry of what diabetes and the implications of having diabetes would mean for them in the long-term. This was linked to the level of information (or lack of) on how DM affects the body, and so some individuals appeared to have a low level of understanding, and as such felt anxious that they would experience problems in the future. Other individuals felt that having **too much** information was not helpful in allaying fears or worries.

#### Guilt

Feelings of guilt were discussed by some of the participants, particularly in the context of blood sugar levels. Participants noted that when blood sugar level readings were high as a result of their behaviour, they often felt guilt and shame. Some individuals who had T1DM, and were diagnosed a number of years ago spoke of guilt associated with particular foods. This was still experienced despite alternative approaches to diabetes self-management which have since become the norm (e.g., feelings of guilt after eating chocolate. Historically individuals with DM were advised not to eat sweet foods. Recently self-management plans allow for consumption of all food types, providing insulin regimes are considered.

#### Stigma

Participants noted that there is a stigma associated with DM. One participant related this to when they were displaying symptoms of a hypoglycaemic episode (commonly referred to as a ‘hypo’). However, most of the comments regarding the stigma of diabetes related to diabetes monitoring and treatment. A number of individuals felt that either monitoring blood sugar levels or administering medication in a public place was not appropriate due to the negative connotations associated with needles. Those individuals altered their own behaviour due to how their actions may be perceived, particularly if children were present.

There was also a feeling that diabetes is negatively portrayed by the media, particularly T2DM. Obesity and poor diet and the media’s portrayal of these in association with diabetes were discussed by some participants. Some individuals went further to discuss how weight and weight management issues were raised by clinicians. They felt there was a misconception that individuals with diabetes had a poor diet or were overweight due to poor decision-making.

#### Control

The term “control” was often used by participants, however it became apparent that the meaning of control was different for different people. Some participants referred to control in the context of *controlling their blood sugar levels*, or *controlling their diabetes* and management of it. For example, individuals discussed keeping control of their diabetes in the context of the monitoring of their blood sugar levels. They would routinely monitor their levels, and perhaps keep a written record of what those readings were. In doing so, they experienced feelings of control, irrespective of what the blood sugar level readings were. Conversely, others spoke of control in that they were able to keep their blood sugar level readings within a given range.

Control was also discussed specifically in the context of self-management. Some individuals felt that they were in control of **their** diabetes as they could control what they chose to eat, how and when to modify daily behaviours, such as what to eat, or when to test blood sugar levels. This could be interpreted that they were more in control of the self-management of their DM, and does not necessarily equate to achieving good control of their blood sugar levels. For some individuals control of behaviours (and possibly blood sugar levels) meant they felt in control of minimising the risk of diabetes-related complications in the future.

#### Hassle

Some individuals noted that having diabetes was a ‘*hassle’.* For some this was related to attending appointments with professionals in order to monitor their DM or diabetes-related complications. Issues such as taking time off work, travelling to and from appointments, parking and so on were problematic (to varying degrees). Others noted having to monitor blood sugar levels was a *hassle* and it was something that had to be fitted in to daily activities/routine. Although it is not a particularly time-consuming task for most, it was “something else” to have to deal with. Participants mentioned that having to carry blood sugar monitoring equipment, and/or food and medication was also a *hassle*.

#### Stress

The term “stress” was frequently used by participants, but its meaning varied between participants. Some noted that there was a degree of stress associated with being diagnosed with DM. For some individuals the stress was associated with uncertainty about the future (what the implications of having diabetes would be for them or how diabetes self-management would impact upon their life). Others spoke of stress relating to managing their diabetes, (planning the day ahead, carrying equipment round etc.). Some participants acknowledged that even routine appointments with health care professionals were a stressful experience. They had concerns over whether they were managing their blood sugar levels correctly, achieving stable and consistent blood sugar levels over a period of time, administering medication correctly, whether their diabetes was “stable” and/or whether there had been any implications of having diabetes on areas of their health (such as neuropathy). Individuals noted that there was a degree of stress with the “review appointments”, and spoke of concerns of being “told off”.

#### Feeling supported

Individuals noted that self-managing DM was positively affected by *feeling supported.* The type of support received (and wanted) varied from one person to another. To some the support came from friends and family, and manifested itself in a variety of ways. These included having someone to go with them to appointments, helping lifestyle changes and choices (such as diet and exercise), being there to talk/listen, reminded about monitoring blood sugar levels or to take medication. Some individuals discussed about the need for support from professionals. The level and meaning of support did vary between respondents. They included issues such as getting regular feedback on performance, up-to-date information on diabetes management, a point of contact for questions, and maintaining motivation. Some individuals acknowledged that regular interaction with professionals/clinicians was important to avoid becoming blasé over DM self-management. For some participants having the knowledge and understanding of diabetes and how to manage the condition was vital in order for them to firstly accept the diagnosis, and then to comply with good diabetes self-management techniques.

#### Freedom

Participants noted that having DM meant that there was a lack of *freedom* in how they chose to live their daily lives. This could be linked to what activities they chose to do on any given day, or what they chose to eat and when they chose to eat it. Either of these components then contributed to individuals having to consider what they may need to take (or have with them) to monitor and manage their blood sugar levels (such as testing kit or insulin). Some participants noted that due to changes in how people are advised to self-manage their diabetes, they have more *freedom*, particularly with respect to food choices.

### Step IV: Selecting attributes and wording of the items for the questionnaire

Attributes reflecting both health related quality of life (HRQoL) and self-management are included in the questionnaire in order to provide a holistic measure. The questionnaire is designed therefore to be used in the economic evaluation of health care interventions for both T1DM and T2DM that reflects both HRQoL and self-management outcomes simultaneously.

#### Health attributes

To identify health attributes for the questionnaire, earlier psychometric work conducted with the Diabetes Health Profile (DHP-1/DHP-18) [12, 13] was used as a starting point. The previous psychometric analyses resulted in a health state classification system amenable to valuation to form a diabetes specific preference-based measure, the DHP-5D. It is based on items from the DHP-18 (mood and barriers to activity) and DHP-1 (fear of hypoglycaemic attacks), supplemented by an item from the SF-6D vitality dimension [12, 13]. The DHP-5D has five dimensions, psychological distress (2 items of mood and social limitations), disinhibited eating, fear of hypoglycaemic attack (four response levels) and vitality (five response levels).

For this study, the content of the DHP-5D was reviewed by the research team against the results of the patient interviews. In order to be able to incorporate more self-management items it was decided to remove the disinhibited eating item. It was felt that this item overlapped with some of the self-management attributes. The wording of the remaining four items was also reviewed and minor revisions were made to the wording of one item to make it compatible with the self-management items.

#### Self-management attributes

All self-management attributes identified from the patient interviews were considered by the research team for inclusion in the questionnaire to go alongside the health attributes. The general guiding principle was that attributes should focus on *how* self-management affects the patient rather than any specific process-related issues.

***Fear/anxiety*** were not included as it was felt they were too vague and in the interviews they were not specifically related to self-management. ***Guilt*** was not included as it is a driver of self-management rather than a consequence of self-management. ***Stigma*** was not included as it was felt not to be about self-management per se. ***Control*** was included with the sense that it is about whether you *feel* in control of your diabetes (rather than whether you *are* actually in control). ***Hassle*** was included as it was clearly identified to be related to self-management. Although the term hassle was used by the interviewees it was noted that this term needed testing with a patient focus group to make sure it is understandable in this population. ***Stress*** was included as it was clearly related to self-management. *Feeling supported* was included but it was noted that there was weaker support for this attribute from the interviews and so this should be tested in a patient focus group as to whether it should be included or not. ***Freedom*** was not included as it was felt to be too closely linked to hassle and control. Therefore four attributes were included (***control***, ***hassle***, ***stress*** and ***support***).

The three attributes from the DHP and energy for the attributes designed to capture HRQoL, and the four attributes identified from the patient interviews cover self-management, resulting in the following eight attributes for inclusion in the questionnaire.

1. Mood
2. Hypoglycaemic attacks
3. Social Limitations
4. Energy
5. Control
6. Hassle
7. Stress
8. Support

### Developing levels for the attributes

Levels for each of the self-management and energy attributes were developed by the research team. The number and wording of levels were informed by the existing severity levels used in the DHP in order to aim for consistency across the questionnaire. Decisions were made about whether attributes were about the severity or frequency of an attribute based on the patient interviews.

### Step V: Testing the draft version of the questionnaire

#### Focus group

The draft descriptive system was taken to a focus group which consisted of 4 diabetes patients (a mixture of T1DM and T2DM) who were asked to comment on the following:

1. The response options of the items (frequency vs. severity)
2. Whether feeling supported should be included as this was the weakest attribute arising from the qualitative research
3. Whether the term hassle was suitable and understandable
4. The overall wording of the questionnaire

Results from the focus group were that the ***control*** attribute was clearly identified to be about severity. Patients commented that the highest level for the ***control*** (severity) question (total control of diabetes) was not really possible. The levels for ***control*** were therefore altered accordingly. There was agreement that the ***support*** dimension should be included, and that the term ***hassle*** was good, clear and understandable. Suggestions were made that ***support*** should include an explanation in brackets that it is about both clinical *and* family support. In response to feedback from the focus group, the descriptive system was subsequently revised.

### Administering the draft questionnaire

The revised questionnaire was piloted online with 15 DM patients. Patients were asked to complete the descriptive system and give general feedback on whether it was clear and understandable. All patients completed all questions. The spread in responses across the levels in the attributes was good. The only amendment to the questionnaire was that it was suggested that the description of ***support*** should be slightly amended to ‘family, friends and health care professionals’. The final questionnaire (Health and Self-Management in Diabetes questionnaire, HASMID version 1) is shown in Table 3. The HASMID^v1^ questionnaire consists of 8-items, each with four response options. Response options are scored from 0 to 3, with a higher score indicating little or no impact upon HRQoL. The overall questionnaire is therefore scored from 0 to 24, with a high score indicating good HRQoL, and a low score indicating poor HRQoL.

**Table 3:** Descriptive system of the HASMID^v1^ questionnaire

Mood	You **never** find yourself losing your temper over small things
	You **sometimes** find yourself losing your temper over small things
	You **usually** find yourself losing your temper over small things
	You **always** find yourself losing your temper over small things
Hypoglycaemic attacks	You **never** worry about going hypo
	You **sometimes** worry about going hypo
	You **usually** worry about going hypo
	You **always** worry about going hypo
Energy	You are **never** tired
	You are **sometimes** tired
	You are **usually** tired
	You are **always** tired
Social Limitations	Your days are **never** tied to mealtimes
	Your days are **sometimes** tied to meal times
	Your days are **usually** tied to meal times
	Your days are **always** tied to meal times
Control	You feel you have **a lot of control** of your diabetes
	You feel you have **some control** of your diabetes
	You feel you have **little control** of your diabetes
	You feel you have **no control** of your diabetes
Hassle	You find your life with diabetes is **never** a hassle
	You find your life with diabetes is **sometimes** a hassle
	You find your life with diabetes is **often** a hassle
	You find your life with diabetes is **always** a hassle
Stress	You find your life with diabetes is **never** stressful
	You find your life with diabetes is **sometimes** stressful
	You find your life with diabetes is **often** stressful
	You find your life with diabetes is **always** stressful
Support (All support you have; from family, friends and health care professionals)	You feel **totally supported** with your diabetes
	You feel you have **a lot of support** with your diabetes
	You feel you have **a little support** with your diabetes
	You feel you have **no support** with your diabetes

## Discussion

This paper describes the steps undertaken to develop HASMID^v1^, a questionnaire that can be used to determine the impact of self-management in diabetes for both T1DM and T2DM. Current measures are limited in their ability to compare the effectiveness of interventions on self-management of diabetes. Clinical outcomes (e.g., HbA1c) are not always appropriate since these fail to measure the impact of the condition upon an individual’s quality of life. Clinical outcome data is often used as an indicator of treatment success or failure, however such measures fail to take into account the patient’s perspective, and cannot capture how a disease (or condition) impacts upon their daily lives. In contrast patient reported outcome measures (PROMs) are self-reported by the patient and take into account the patient’s perspective. Recently there has been an increasing demand for PROMs both within routine clinical practice and research. Good PROMs must demonstrate key properties, such as reliability, validity and sensitivity. Current guidance advocates instrument developers to make PROM instruments and related development history available and accessible publically [14]. However, the literature on the development of descriptive systems for PROMs is sparse in comparison to the assessment of the psychometric properties of the instrument itself. This paper presents the development of an instrument that includes questions on the impact of self-management from the patient’s perspective.

Many legacy PROMs were developed by collating a list of potential attributes (questions) from existing instruments and expert opinion. In contrast “bottom-up” methodologies are being increasingly used in the development of other PROMs for both generic and condition-specific instruments [15–17]. It is argued that by using qualitative data techniques pertinent issues from the patient’s perspective are identified. This study adopted a mixed-methods approach to inform the content of the descriptive system. A conceptual model of self-management in T1DM and T2DM was derived following the literature review and interviews with patients. Item generation was based upon the conceptual model, with inclusion of existing health attributes that have been tested within the target population (items from the DHP-5D). An important consideration during the development of the questionnaire was to ensure the instrument would be suitable for both T1DM and T2DM individuals. Treatment-specific items were not considered as this could limit the use of the instrument in evaluating self-management interventions across patient groups. Further refinement of the instrument was achieved through cognitive debriefing, incorporating patients’ views within each aspect of the development phase, ensuring good content and face validity. The rigorous and transparent approaches undertaken to develop the HASMID^v1^ questionnaire emphasises its usefulness as a PROM.

The study is not without its limitations. The qualitative phase included a sample that covered the main age groups, type of diabetes, and duration of diabetes. However, the sample was not balanced with respect to ethnicity. Out of the 32 participants interviewed, only 4 participants were non-white. Due to time and resource limitations it was not possible to explore whether any cultural differences exist with respect to self-management of diabetes. Further work is needed to determine whether there are any additional items that may be relevant in a more diverse population. The items assessing health were taken from an existing measure (DHP) and were generated separately to the self-management items.

Further work is ongoing to formally assess the psychometric properties of the HASMID^v1^ measure using a large patient sample.

## Conclusion

This paper has described the development of a questionnaire for self-management and health outcomes in diabetes for both T1DM and T2DM. The HASMID^v1^ questionnaire is a short, easy-to-complete PROM. It has been developed following a series of rigorous iterations, with high involvement of patients and service-users to ensure good face validity. Further work is currently being undertaken to assess the performance of the HASMID^v1^ questionnaire in an independent sample, and to examine aspects of reliability, such as the responsiveness (sensitivity to change) of the instrument.

## Additional file

Additional file 1: Appendices 1-3. (DOCX 24 kb)
