# Supplementary material for: Developing a questionnaire to determine the impact of self-management in diabetes: giving people with diabetes a voice
Source: Health Qual Life Outcomes. 2017 Jul 18;15:146. doi: 10.1186/s12955-017-0719-4 (PMC5516314; doi:10.1186/s12955-017-0719-4)
Supplement: Additional file 1: — Appendices 1-3. (DOCX 24 kb) [file 12955_2017_719_MOESM1_ESM.docx]

**Additional file 1**

**Appendix 1 Search Strategy**

1. **Filters**

- SIGN Systematic Reviews filter for Medline, Embase, Cinahl: <http://www.sign.ac.uk/methodology/filters.html#systematic>
- Health Evidence Bulletins Wales for PyscINFO: <http://hebw.cf.ac.uk/methodology/appendix2.htm> (scroll down to SEARCH FILTER FOR PSYCHLIT/CLIN PSYCH/PSYCHINFO at bottom of page; steps 1-17 is the reviews filter)

1. **Strategies**

**Medline strategy (searched 02/05/2014)**

1 *Diabetes Mellitus, Type 2/ or *Diabetes Mellitus, Type 1/ (108942)

2 diabet*.ti. (236220)

3 1 or 2 (254563)

4 *Self Care/ (12169)

5 (self manag* or self-manag*).ti. (3103)

6 (self care or self-care).ti. (2821)

7 4 or 5 or 6 (13716)

8 3 and 7 (2387)

9 Meta-Analysis/ (47464)

10 meta analy$.tw. (62349)

11 metaanaly$.tw. (1306)

12 meta analysis.pt. (47464)

13 (systematic adj (review$1 or overview$1)).tw. (53241)

14 exp Review Literature/ (1871786)

15 or/9-14 (1926042)

16 cochrane.ab. (30264)

17 embase.ab. (28910)

18 (psychlit or psyclit).ab. (891)

19 (psychinfo or psycinfo).ab. (12120)

20 (cinahl or cinhal).ab. (10160)

21 science citation index.ab. (1992)

22 bids.ab. (365)

23 cancerlit.ab. (581)

24 or/16-23 (51967)

25 reference list$.ab. (9783)

26 bibliograph$.ab. (11632)

27 hand-search$.ab. (3956)

28 relevant journals.ab. (725)

29 manual search$.ab. (2349)

30 or/25-29 (25437)

31 selection criteria.ab. (19766)

32 data extraction.ab. (9808)

33 31 or 32 (27997)

34 review.pt. (1867886)

35 33 and 34 (18425)

36 comment.pt. (581335)

37 letter.pt. (837925)

38 editorial.pt. (353561)

39 animal/ (5290661)

40 human/ (13387877)

41 39 not (39 and 40) (3837921)

42 or/36-38,41 (5113819)

43 15 or 24 or 30 or 35 (1939626)

44 43 not 42 (1778142)

45 8 and 44 (235)

46 limit 45 to (english language and yr="2008 -Current") (110)

**Embase strategy (searched 02/05/2014)**

1 *diabetes mellitus/ (172194)

2 diabet*.ti. (302408)

3 1 or 2 (344299)

4 *self care/ (10442)

5 (self manag* or self-manag*).ti. (3921)

6 (self care or self-care).ti. (3315)

7 4 or 5 or 6 (12543)

8 3 and 7 (2351)

9 exp Meta Analysis/ (77997)

10 ((meta adj analy$) or metaanalys$).tw. (79546)

11 (systematic adj (review$1 or overview$1)).tw. (63854)

12 or/9-11 (150488)

13 cancerlit.ab. (640)

14 cochrane.ab. (35305)

15 embase.ab. (33310)

16 (psychlit or psyclit).ab. (941)

17 (psychinfo or psycinfo).ab. (8497)

18 (cinahl or cinhal).ab. (10904)

19 science citation index.ab. (2100)

20 bids.ab. (448)

21 or/13-20 (55721)

22 reference lists.ab. (9554)

23 bibliograph$.ab. (14705)

24 hand-search$.ab. (4508)

25 manual search$.ab. (2711)

26 relevant journals.ab. (811)

27 or/22-26 (29094)

28 data extraction.ab. (11663)

29 selection criteria.ab. (19185)

30 28 or 29 (29705)

31 review.pt. (1942963)

32 30 and 31 (15843)

33 letter.pt. (841043)

34 editorial.pt. (446718)

35 animal/ (1563694)

36 human/ (14564825)

37 35 not (35 and 36) (1186385)

38 or/33-34,37 (2460532)

39 12 or 21 or 27 or 32 (185010)

40 39 not 38 (178648)

41 8 and 40 (80)

42 limit 41 to (english language and yr="2008 -Current") (49)

**CINAHL strategy (searched 02/05/2014)**

| S23 | S20 and S21 and S22 | **Limiters** - Published Date: 20080101-20121231  **Search modes** - Boolean/Phrase |
| --- | --- | --- |
| S22 | LA English | **Limiters** - Published Date: 20080101-20121231  **Search modes** - Boolean/Phrase |
| S21 | DT 20080101-20140501 | **Limiters** - Published Date: 20080101-20121231  **Search modes** - Boolean/Phrase |
| S20 | S8 and S19 | **Search modes** - Boolean/Phrase |
| S19 | S15 or S16 or S17 or S18 | **Search modes** - Boolean/Phrase |
| S18 | (MH "Animals") | **Search modes** - Boolean/Phrase |
| S17 | PT Editorial | **Search modes** - Boolean/Phrase |
| S16 | PT Letter | **Search modes** - Boolean/Phrase |
| S15 | PT Commentary | **Search modes** - Boolean/Phrase |
| S14 | S9 or S10 or S11 or S12 or S13 | **Search modes** - Boolean/Phrase |
| S13 | TI ( (systematic N1 (review or overview)) ) OR AB ( (systematic N1 (review or overview)) ) | **Search modes** - Boolean/Phrase |
| S12 | (MH "Literature Review+") | **Search modes** - Boolean/Phrase |
| S11 | TI Metaanaly* OR AB Metaanaly* | **Search modes** - Boolean/Phrase |
| S10 | TI Meta analys* OR AB Meta analys* | **Search modes** - Boolean/Phrase |
| S9 | (MH "Meta Analysis") | **Search modes** - Boolean/Phrase |
| S8 | S3 and S7 | **Search modes** - Boolean/Phrase |
| S7 | S4 or S5 or S6 | **Search modes** - Boolean/Phrase |
| S6 | TI self care OR TI self-care | **Search modes** - Boolean/Phrase |
| S5 | TI self manag* OR TI or self-manag* | **Search modes** - Boolean/Phrase |
| S4 | (MM "Self Care") | **Search modes** - Boolean/Phrase |
| S3 | S1 OR S2 | **Search modes** - Boolean/Phrase |
| S2 | TI diabet* | **Search modes** - Boolean/Phrase |
| S1 | (MM "Diabetes Mellitus, Type 2") OR (MM "Diabetes Mellitus, Type 1") | **Search modes** - Boolean/Phrase |

**Cochrane strategy (searched 02/05/2014)**

#1 MeSH descriptor: [Diabetes Mellitus, Type 1] this term only

#2 MeSH descriptor: [Diabetes Mellitus, Type 2] this term only

#3 diabet*:ti

#4 (or #1-#3)

#5 MeSH descriptor: [Self Care] this term only

#6 (self manag* or self-manag*):ti

#7 (self care or self-care):ti

#8 (or #5-#7)

#9 #4 and #8 Publication Date from 2008 to 2014, in Other Reviews and Technology Assessments

**Web of Science Strategy (searched 06/05/2014)**

| # 7 | [**34**](http://apps.webofknowledge.com/summary.do?product=WOS&doc=1&qid=21&SID=P2o6LelPKZLVOElXc4H&search_mode=Refine) | #1 AND #4  **Refined by:** **DOCUMENT TYPES:** ( REVIEW ) AND **LANGUAGES:** ( ENGLISH )  *Indexes=SCI-EXPANDED, SSCI Timespan=2008-2014* |
| --- | --- | --- |
| # 6 | [**35**](http://apps.webofknowledge.com/summary.do?product=WOS&doc=1&qid=20&SID=P2o6LelPKZLVOElXc4H&search_mode=Refine) | #1 AND #4  **Refined by:** **DOCUMENT TYPES:** ( REVIEW )  *Indexes=SCI-EXPANDED, SSCI Timespan=2008-2014* |
| # 5 | [**881**](http://apps.webofknowledge.com/summary.do?product=WOS&doc=1&qid=19&SID=P2o6LelPKZLVOElXc4H&search_mode=AdvancedSearch) | #1 AND #4  *Indexes=SCI-EXPANDED, SSCI Timespan=2008-2014* |
| # 4 | [**4,577**](http://apps.webofknowledge.com/summary.do?product=WOS&doc=1&qid=18&SID=P2o6LelPKZLVOElXc4H&search_mode=AdvancedSearch) | #2 OR #3  *Indexes=SCI-EXPANDED, SSCI Timespan=2008-2014* |
| # 3 | [**1,994**](http://apps.webofknowledge.com/summary.do?product=WOS&doc=1&qid=17&SID=P2o6LelPKZLVOElXc4H&search_mode=AdvancedSearch) | TI=(self care OR self-care)  *Indexes=SCI-EXPANDED, SSCI Timespan=2008-2014* |
| # 2 | [**2,854**](http://apps.webofknowledge.com/summary.do?product=WOS&doc=1&qid=12&SID=P2o6LelPKZLVOElXc4H&search_mode=AdvancedSearch) | TI=(self manag* OR self-manag*)  *Indexes=SCI-EXPANDED, SSCI Timespan=2008-2014* |
| # 1 | [**89,943**](http://apps.webofknowledge.com/summary.do?product=WOS&doc=1&qid=8&SID=P2o6LelPKZLVOElXc4H&search_mode=AdvancedSearch) | TI=(diabet*)  *Indexes=SCI-EXPANDED, SSCI Timespan=2008-2014* |

**PsychINFO strategy (searched 06/05/2014)**

1 *Diabetes/ or *Diabetes Mellitus/ (9403)

2 diabet*.ti. (8767)

3 1 or 2 (10099)

4 (self manag* or self-manag*).ti. (2045)

5 (self care or self-care).ti. (1258)

6 4 or 5 (3293)

7 3 and 6 (612)

8 meta analysis.sh. (3381)

9 meta-anal:.tw. (18437)

10 metaanal:.tw. (384)

11 meta analysis.id. (3538)

12 (systematic: and (review: or overview)).tw. (22885)

13 (critical: and apprais:).tw. (2796)

14 (critical: and review:).tw. (30720)

15 8 or 9 or 10 or 11 or 12 or 13 or 14 (68447)

16 literature review.sh. (21921)

17 literature review.id. (19247)

18 16 or 17 (22481)

19 15 or 18 (88347)

20 case report.sh. (22453)

21 19 not 20 (88199)

22 7 and 21 (39)

23 limit 22 to human (39)

24 limit 23 to (english language and yr="2008 -Current") (22)

**Appendix 2** **List of potential themes to be discussed with patients with diabetes**

| Lack of education | Lack of needed skills |
| --- | --- |
| Telehealth | Adherence |
| Self-care behaviours | Taking medication |
| Monitoring | Diet |
| Exercise | Reinforcement |
| Healthy eating | Being active |
| Problem solving | Reducing risks |
| Healthy coping | Getting feedback on performance |
| Information on consequences of behaviour | Barrier identification/problem solving |
| Focussing on past success | Agree behavioural contract |
| Fear arousal (hypos) | Depression |
| Distress | Psychological well-being |
| Self-efficacy | Stress |
| Communication | Action planning |
| Rewards | Prompts/cues |
| Emotional control | Regret |
| Individual face-to-face | Group face-to-face |
| Phone based | DVD based |
| Internet based | Video conferencing |
| Number of sessions | IT/web-based/phones |
| Satisfaction | Knowledge |
| Empowerment | Health literacy |
| Memory | Rationale for SM |
| Information overload | Health beliefs (HBM) |
| Social support | Financial support |
| Education/advice | Appraisal – feedback |
| Emotional support | Self-efficacy (self-belief) |
| Coping | Managing stress |
| Denial | Planning |
| Loss of control | Fatigue |
| Loss of interest | Reduced decision-making ability |
| Alcohol consumption | Family support and partner support |
| Location (urban vs. rural) | Foot care |
| Requiring insulin – failure | Fear about dose adjustment |
| Daily SM compromising adherence | Self-confidence |
| Interpreting food labels | Family support |
| Marital satisfaction/stress | Family traditions and cultural issues (food) |
| Individual acceptability | Learning experience |
| Physical factors | Personal factors |
| Access factors | Care factors |

**Appendix 3 Topic Guide for interviews**

**Introduction**

- Name and aim of study
- Confidentiality
- Recording

**Themes**

- Task-related
  - Monitoring and burden – individually and separate
  - Information overload
  - Satisfaction
  - Reinforcement of behaviour
  - Getting feedback on performance/satisfaction
  - Self-efficacy
  - Problem solving
- Physical
  - Exercise
  - Healthy eating
  - Being active
- Emotional
  - Stress
  - Distress
  - Denial
  - Acceptance
  - Regret/guilt
  - Confidence
  - Coping
  - Control
  - Empowerment – flip side – reduced decision-making ability
  - Requiring insulin – failure
  - Blame – themselves/others/access to services
  - Self-image
- Social
  - Support
  - Relationship stress
  - Peer pressure
  - Understanding of others – friends/family/colleagues
